# In Vitro and In Planta Antagonistic Effect of Endophytic Bacteria on Blight Causing *Xanthomonas axonopodis* pv. *punicae*: A Destructive Pathogen of Pomegranate

**DOI:** 10.3390/microorganisms11010005

**Published:** 2022-12-20

**Authors:** Nripendra Vikram Singh, Jyotsana Sharma, Manjushri Dinkar Dongare, Ramakant Gharate, Shivkumar Chinchure, Manjunatha Nanjundappa, Shilpa Parashuram, Prakash Goudappa Patil, Karuppannan Dhinesh Babu, Dhananjay Morteppa Mundewadikar, Unnati Salutgi, Muskan Tatiya, Aundy Kumar, Rajiv Arvind Marathe

**Affiliations:** 1 ICAR-National Research Centre on Pomegranate, Kegaon, Solapur 413255, India; 2 Lokmangal College of Agricultural Biotechnology, Solapur 413222, India; 3 Walchand College of Arts and Science, Solapur 413006, India; 4 ICAR-Indian Agricultural Research Institute, New Delhi 110012, India

**Keywords:** bacterial blight disease, endophytes, pomegranate, *Xanthomonas axonopodis* pv. *punicae*, host plant response

## Abstract

Pomegranate bacterial blight caused by *Xanthomonas axonopodis* pv. *punicae* (*Xap*) is a highly destructive disease. In the absence of host resistance to the disease, we aimed to evaluate the biocontrol potential of endophytic bacteria against *Xap*. Thus, in this study, we isolated endophytes from pomegranate plants, identified them on the basis of 16S rDNA sequencing, tested them against *Xap*, and estimated the endophyte-mediated host defense response. The population of isolated endophytes ranged from 3 × 10^6^ to 8 × 10^7^ CFU/g tissue. Furthermore, 26 isolates were evaluated for their biocontrol activity against *Xap*, and all the tested isolates significantly reduced the in vitro growth of *Xap* (15.65% ± 1.25% to 56.35% ± 2.66%) as compared to control. These isolates could reduce fuscan, an uncharacterized factor of *Xap* involved in its aggressiveness. Lower blight incidence (11.6%) and severity (6.1%) were recorded in plants sprayed with endophytes 8 days ahead of *Xap* spray (Set-III) as compared to control plants which were not exposed to endophytes (77.33 and 50%, respectively%) during in vivo evaluation. Moreover, significantly high phenolic and chlorophyll contents were estimated in endophyte-treated plants as compared to control. The promising isolates mostly belonged to the genera *Bacillus*, *Burkholderia*, and *Lysinibacillus*, and they were deposited to the National Agriculturally Important Microbial Culture Collection, India.

## 1. Introduction

Pomegranate (*Punica granatum* L.) is one of the oldest fruits known to mankind [1]. The genus *Punica* belongs to the family Lythraceae, order Myrtales, and it is composed of two species: *P*. *protopunica* Balf. and *P*. *granatum* L. (2n = 2x = 16), of which *P*. *granatum* L. is mainly cultivated for fruit production [2]. Its fruits are a rich source of antioxidants and minerals such as calcium, iron, and sulfur [3,4]. This fruit crop has abundant health promoting properties and is one of the richest sources of phenolic compounds including ellagitannins (ETs), flavonols, and flavonoids with potential antioxidant, anticancer, and anti-atherosclerotic properties [5,6].

Pomegranate is grown primarily in climatically and edaphically challenged areas with water scarcity and harsh climatic conditions prevailing for a considerable period in the growing season. It is widely cultivated in drier parts of Southeast Asia, China, Turkey, Egypt, Spain, Israel, Japan, the USA (California), the West Indies, tropical America, Iran, and India [7,8]. India is the global leader in pomegranate acreage and production with 288 thousand ha area and 3271 thousand tons of annual production (www.agricoop.nic.in (accessed on 28 September 2022)). The high returns on investment, versatile adaptability, abundant nutraceutical, therapeutic, and nutritional value, impressive range of value-added products, and high export demand have resulted in the enhanced popularity of this crop among growers and consumers alike [9,10,11]. Thus, it is an ideal crop for replacing subsistence farming and alleviating the livelihood and nutritional security of farmers in water-scarce regions of the world.

However, pomegranate growers of India have received a setback due to a severe outbreak of bacterial blight disease caused by *Xanthomonas axonopodis* pv. *punicae* in recent times, resulting in severe yield losses to the tune of 60–80% [12,13]. The strain reported to cause severe bacterial blight in central India and other parts of the country was recently characterized and found to be of a clonal lineage [14]. The pomegranate bacterial blight has also been reported in Pakistan, Turkey, and South Africa [15,16,17]. The management of bacterial blight disease by resorting only to the use of expensive chemicals and antibiotics/bactericides is neither economically viable nor environmentally sustainable. Thus, the utilization of endophytes may be explored and subsequently intensified as one of the ecofriendly approaches for the management of bacterial blight.

Endophytic microbes can be isolated from surface-disinfected plant tissues or extracted from within the plant, as the endosphere and the microenvironment of plant shoots, leaves, and roots harbor these microbes [18,19,20]. Interestingly, endophytes are known to influence the physiological and biochemical functioning of the host plant and are reported to have a significant impact on growth promotion, resistance reactions, and overcoming abiotic stresses [21,22,23,24]. The beneficial interaction of these microbes with the host plant may be explored as an efficient method for biological control of pathogens for sustainable crop production [21]. In order to exploit the potential benefits of endophytes in managing plant diseases, systematic research investigations need to be planned to understand the mechanisms involved in host endophyte interactions and their regulation in response to biotic and abiotic factors, thus optimizing colonization conditions and in-depth investigations of population dynamics [25,26]. The present investigation was carried out to isolate and identify bacterial endophytes from pomegranate plant parts and evaluate their in vitro antibacterial activity against *Xanthomonas axonopodis* pv. *punicae.* In addition, in planta experiments were also conducted to evaluate the potential of endophytes in reducing blight incidence. The results of the study indicate that these endophytes can be successfully utilized for ecofriendly management of bacterial blight disease in pomegranate.

## 2. Materials and Methods

### 2.1. Experimental Site

The present experiment was carried out during the period 2017–2021 at the Tissue Culture and Biotechnology Facility, Plant Pathology Laboratory, and Polyhouses of the ICAR National Research Center on Pomegranate (NRCP), Solapur, India, having 17°43′ N latitude, 75°50′ E longitude, and 483.5 m altitude above mean sea level.

### 2.2. Isolation of Bacterial Endophytes from Field Grown Plants

The leaves, roots, and stems collected from four different varieties/genotypes (Bhagawa, Ganesh, Nana and *Daru*) of 8 year old field-grown pomegranate healthy plants were used for the isolation of endophytic bacteria. Collected samples (leaves, roots, and stems) were washed thrice in sterile distilled water. Approximately one gram of sample was weighed separately, cut into small segments (2–3 cm), and surface-sterilized with a 2.0% solution of sodium hypochlorite (4.0% a.i. *w/v*) for 10 min [27]. Then, plant parts were rinsed with sterile distilled water and treated with 70% alcohol for 2 min followed by four washes with sterile distilled water. The efficacy of sterilization was checked by the tissue imprint method on nutrient agar (NA) and King’s B agar plates [28]. Briefly, an imprint of the surface sterilized tissue was made on the medium by gently pressing it on the surface of solidified medium. The plates were then incubated at the optimum temperature to check for any microbial growth. Since the tissue had been properly sterilized, no colonies appeared on the imprinted plate. Similarly, a 0.1 mL aliquot from the final wash was plated on nutrient agar (NA) and King’s B agar plates, and no colonies were observed. The sterilized samples were macerated to homogenize under aseptic conditions with a sterile mortar and pestle in 9.0 mL of phosphate-buffered saline [19]. The mixture was serially diluted to 10^−5^, and each dilution (10^−1^ to 10^−5^) was spread on NA and King’s B agar media with three replications. The plates were incubated at 28 °C for 48–72 h. The well-isolated colonies from each plate were selected and sub-cultured on NA medium. The representative isolates were preserved at −80 °C in 20% glycerol stock and nutrient glucose broth (NGB; peptone, beef extract powder, and dextrose) for further studies. In total, 14 bacterial endophytes were isolated from different tissues of pomegranate plants (EB1–EB14).

### 2.3. Isolation of Endophytes from In Vitro Grown Explants

First, 3–4 cm long and 1 month old nodal segments or fresh shoot tips were washed three times with tap water followed by treatment with the solution of 0.2% Carbendazim-50% WP + 0.2% [Metalaxyl (4%) + Mancozeb (64%)] + 0.05% 2-bromo-2-nitropropane-1,3-diol for 30 min and rinsing twice with autoclaved water (sterilized at 121 °C and 15 psi for 1 h). The explants were then surface-sterilized 2.0% solution of sodium hypochlorite (4.0% a.i. *w/v*) for 10 min and washed three times with autoclaved water. The explants were inoculated in a modified MS medium supplemented with 0.5 mg/L 6-benzyl adenine purine (BAP, HiMedia^TM^ make) and 0.1 mg/L naphthalene acetic acid (NAA, HiMedia^TM^ make). After 15–20 days of inoculation, the microbial population started oozing out from the cut end of explants in the culture medium and the whole mass of medium in and around cut end of the explants having the bacterial ooze was taken directly as a source of endophyte (~0.3 g media mass) and serially diluted up to 10^−5^. Each dilution (10^−1^ to 10^−5^) of endophytes was inoculated on Nutrient Glucose Agar medium (NGA; peptone, beef extract powder, dextrose, and agar) with three replications. The plates were incubated at 28 °C for 48–72 h. The well-isolated colonies from each plate were selected and sub-cultured on a NA medium. The representative isolates were preserved at −80 °C in 20% glycerol stock and NGB for further studies. Twelve bacterial endophytes were isolated from in vitro established cultures (TC-1, TC-2, TC-4, TC-6, TC-7, TC-9, TC-10, TC-130, TC-310, TC-137, TC-A_2_B, and TC-B).

### 2.4. In Vitro Evaluation of Endophytes against Xap

In vitro screening for bacterial antagonism of endophytes was performed using a dual-culture confrontation assay in Petri plates. The inoculum of bacterial endophytes and the pure culture of pathogen *X. axonopodis* pv. *punicae* were multiplied on NGA for 72 h at 28 ± 1 °C. The 5 mm circular disc of endophyte was placed in the center, and the pathogen *X. axonopodis* pv. *punicae* was streaked in a square fashion around the antagonist at around 20 mm distance from center of the plate. The plates were incubated at 28 ± 1 °C and observations on *X. axonopodis* pv. *punicae* growth was taken from third day until the eighth day. The thickness of *Xap* growth was measured physically, which indicates the growth of *Xap* under the influence of endophytes and compared with *Xap* growth in control plate (NGA inoculated with *Xap* without any endophyte). Since *Xap* was inoculated in a square pattern with the endophyte in the center, the growth included the size of the four sides of the square. Hence, the mean of the four sides was used, and data were recorded for three replicates per treatment (*n* = 3). The effective endophytes were rescreened for confirmation.

### 2.5. Challenge Inoculation and In Vivo Evaluation of Endophytes against Xap

A total of 10 promising bacterial endophytes were selected on the basis of the results of in vitro *Xap* inhibition assays. The best-performing isolates from EB (*n* = 4) and TC (*n* = 6) series were tested in pot culture trials. The 6 month old pomegranate air-layered plants were used for the study. The plants were exposed to high humidity (more than 95%) for 24 h before inoculation and 48 h after inoculation [29]. A full loop of the pure culture of *Xanthomonas axonopodis* pv *. punicae* maintained on NGA was inoculated in Nutrient Glucose Broth (NGB; peptone, beef extract powder, and dextrose) and incubated on a shaker at 100 rpm. After 48–72 h of growth of *Xap* in broth, the bacterial suspension was diluted to 10^7^ to 10^8^ cells per mL (OD_600 nm_ = ca. 0.2–0.3). The spray inoculation method was used for challenge inoculation, and about 25 mL of bacterial suspension solution was sprayed on each pomegranate plant to induce infection [29]. The polyhouse temperature was maintained at 28 ± 1 °C and RH above 50% during the study.

Similarly, endophytes were also inoculated in NGB and incubated on a shaker at 100 rpm. After 24–48 h of growth of endophytes in broth, the bacterial suspension was diluted to 10^7^ to 10^8^ cells per ml (OD_600 nm_ = ca. 0.2–0.3). About 25 mL of endophyte suspension solution was sprayed on each plant using one of the following three strategies:

1. Set-I, where *Xap* and endophyte culture broths were sprayed at the same time.
2. Set-II, where *Xap* was sprayed 8 days earlier than endophytes.
3. Set-III, where endophyte culture broths were sprayed 8 days earlier than the *Xap.*

Plants in the control sets were inoculated with the pathogen only, and no endophyte treatment was given. There were three replications in each treatment and three plants in each replication. A negative control was kept where only sterile water was sprayed.

### 2.6. Reconfirmation of X. axonopodis pv. Punicae Identity Isolated from Challenged Inoculated Host Plants

Symptoms developed on tissues after challenge inoculation were confirmed visually, and the identity of the pathogen was confirmed through *gyrB*-specific PCR-based detection [30]. The genomic DNA of *X. axonopodis* pv. *punicae* was extracted from the *Xap*-infected portion of leaves, and PCR amplification was carried out using *gyrB*-specific primers [30].

### 2.7. Estimation of Disease Incidence and Severity

The percentage disease incidence was calculated using the formula given below.

$$Disease\;incidence\;\left(\%\right)=\frac{Total\;number\;of\;infected\;leaves}{Total\;number\;of\;leaves\;examined}\times 100.$$

The disease severity was calculated using the formula given below [29,31].

$$Disease\;Severity\;\left(\%\right)=\frac{\Sigma \left(\mathrm{Number}\;\mathrm{of}\;\mathrm{leaves}\;\times \;\mathrm{Severity}\;\mathrm{grade}\right)\times 100}{T\times Maximum\;grade},$$

where *T* is the total number of observations.

The severity grade can be visually calculated as a function of the percentage area (leaf or fruit) showing/covered with blight symptoms. It was allotted values of 0–5: 0 = no disease infection, 1 = 1–10% area infected, 2 = 11–25% area infected, 3 = 26–50% area infected, 4 = 51–75% area infected, and 5 = 76–100% area infected [31,32,33].

### 2.8. 16S rDNA-Based Identification of Endophytes and Submission to the National Agriculturally Important Microbial Culture Collection

Genomic DNA was isolated from various endophytes by adopting the modified CTAB method [34]. Universal primers, 27F 5′–AGAGTTTGATCCTGGCTCAG–3′ and 1492R 5′–GGTTACCTTGTTACGACTT–3′ were used for 16S rDNA amplification [35]. The total PCR reaction volume was 50 μL (1× PCR buffer, MgCl_2_ 1.5 mM, each dNTP 50 μM, 5 pmol of each primer, 100 ng of template DNA, and 1 U of TaqDNA polymerase), and the reaction volume was subjected to amplification with PCR conditions set at 96 °C for 2 min of initial denaturation; the amplification was carried out for 35 cycles with denaturation at 94 °C for 30 s, annealing at 60 °C for 1 min, extension at 72 °C for 1 min, and final extension for 10 min at 72 °C. The amplicons were resolved on 1.0% agarose gel and purified using the Gel Elution kit according to the manufacturer’s instructions (Sigma Aldrich, St. Louis, MO, USA). Twenty nanograms of purified amplicon was taken for cycle sequencing reaction using ABI PRISM BigDye Terminators v.1.1 cycle sequencing kit as per the manufacturer’s instructions (Applied Biosystems). To obtain complete coverage of the gene sequence, bidirectional sequencing of the purified products was carried out followed by sequence editing, end trimming, and contig assembly using Vector NTi. The assembled contigs were compared with GenBank sequences by BLASTn analysis. Nucleotide sequence homology was determined using the NCBI databases and the bacterial identity was established by the closest match [36]. The promising endophytic cultures were deposited to the National Agriculturally Important Microbial Culture Collection (NAIMCC), Mau, India, and some of them were also assigned the accession numbers.

### 2.9. Physiological and Biochemical Analysis of Host Pomegranate Plants

The relative water content (RWC) in leaves was determined using the method suggested by Weatherly [37]. Fresh weight of these leaves was measured and then floated overnight on distilled water in a Petri dish. These leaves were then surface-dried by pressing between two blotting papers, and the saturated weight of these leaves was recorded. After that, the samples were dried in an oven at 70 °C until they showed no change in their weight after two consecutive dryings.

$$RWC\;\left(\%\right)=\frac{Fresh\;wight-Oven\;dry\;weight}{Turgid\;weight-Oven\;dry\;weight}\times 100.$$

The total phenols were estimated using the method standardized by Malik and Singh [38]. Shoot tips were used instead of buds, and the values were expressed as catechol equivalent. The Soil Plant Analysis Development (SPAD-502, KONICA MINOLTA) chlorophyll meter was used to estimate relative leaf greenness. Fully matured green leaves were used to analyze the total chlorophyll content [39]. All observations were recorded 90 days after spraying the endophytes.

### 2.10. Statistical Analysis

Data for three replications was recorded for each treatment. Observations for *Xap* inhibition by endophytes were recorded as a function of the reduction in *Xap* growth in vitro and symptoms on leaf tissues in planta. In both cases, inhibition of *Xap* by endophytes was estimated compared to the control. In vitro inhibition data were transformed (arc-sin transformation) since non-transformed data showed large variation. Various physiological and biochemical observations were recorded using three replications per treatment with each replication having three plants. One-way analysis of variance (one-way ANOVA) was carried out, and the critical difference (CD) was expressed at *p* ≤ 0.05. In vitro production of a typical brownish pigment, fuscan, by *Xap* was recorded on a 0–3 scale, where 0 corresponds to no fuscan production and 3 represents heavy fuscan production compared to the control.

## 3. Results

### 3.1. Endophyte Isolation

Using the leaf, stem, and roots of field-grown pomegranate plants and in vitro established nodal segments and shoot tips, as many as 26 bacterial endophytes were isolated. The population of endophytes was estimated, and it ranged from 3 × 10^6^ to 8 × 10^7^ CFU/g of tissue depending on the plant tissue (Table 1).

### 3.2. In Vitro Evaluation of Endophytes against Xap

Bacterial endophytes were screened for their antagonistic activity against *Xap* on NGA medium. The bacterial endophyte isolates from field grown pomegranate plants (EB series) inhibited the growth of *Xap* from 25.03% ± 0.97% to 42.42% ± 1.48%, and bacterial endophytes isolated from in vitro established pomegranate explants (TC series) inhibited the *Xap* growth from 15.65% ± 1.25% to 56.35% ± 2.66%. Within their respective categories, EB9 and TC-6 were found to be the most effective bacterial endophytes with maximum percentage inhibition of *Xap* growth. A dual culture of *Xap* with endophytes resulted in no fuscan production by *Xap* in the medium having EB1, 3, 4, 5, 7, or 13 and TC-6, 137, 310, or A_2_B (Table 2). Sixteen bacterial endophytes resulted in a reduction in or complete inhibition of fuscan production *Xap.*

### 3.3. In Vivo Evaluation of Selected Endophytes against Xap

**Figure 1:**
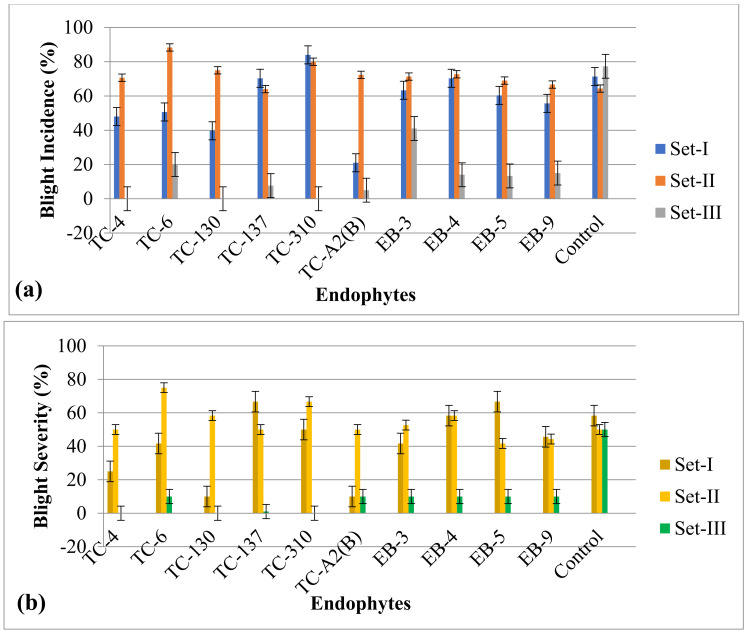
In vivo evaluation of endophytes against bacterial blight in pomegranate cv. Bhagawa in terms of (**a**) disease incidence and (**b**) disease severity. Pomegranate plants were inoculated with pathogen (*Xap*) and endophytes in the following ways: Set-I, *Xap* and endophytes culture broths were sprayed at the same time; Set-II, *Xap* was sprayed 8 days earlier than endophytes; Set-III, endophyte culture broths were sprayed 8 days earlier than the *Xap*. Control plants were challenged only with *Xap*. Error bars are based on the standard error.

In a pot culture experiment, the endophytes showed a significant effect on the reduction in blight incidence as compared to positive control plants (plants challenged inoculated with only *Xap*). The incidence of bacterial blight on leaves of ‘Bhagawa’ plant ranged from 21% (TC-A_2_B) to 71.33% (control), and severity ranged from 10% (TC-130 and TC-A_2_B) to 66.67% (EB-5 and TC-137) in Set-I. When *Xap* and endophyte were both sprayed together (Set-I), the average blight incidence and severity were 56.33% and 41.57%, respectively but the average blight incidence rose to 73% and severity rose to 54.70% when *Xap* was sprayed 8 days ahead of the endophytes (Set-II). However, when endophytes were sprayed 8 days ahead of *Xap* spray (Set-III), then the average incidence was reduced to 11.6% and severity was reduced to 6.1% (Figure 1). The third strategy where endophytes were sprayed 8 days ahead of *Xap* proved to be significantly effective in reducing blight incidence and severity as compared to control plants.

The identity of the *Xap* pathogen was confirmed by reisolating and culturing the *Xap* bacteria from the symptomatic portion of the leaf of positive control plants and amplification of a 491 bp amplicon using *gyrB*-specific primers for PCR amplification of *Xap* DNA (Supplementary Figure S1).

### 3.4. Identification of Promising Endophytes

Promising endophytic bacterial isolates were identified by 16S rDNA sequence comparison with the NCBI GenBank Database (Table 3). Five isolates were identified as belonging to different species of *Bacillus,* TC 7 was identified as *Burkholderia stabilis*, EB6 was identified as *Lysinibacillus macrolides*, and TC 137 was putatively identified as a *Bacillus* species on the basis of biochemical reactions (Table 3).

### 3.5. Host Pomegranate Plant Response to External Application of Promising Endophytes

Pomegranate plants responded significantly to external application of endophytic sprays with enhanced total phenolic and total chlorophyll content of leaves of the host plants as compared to non-inoculated control plants. Total phenolic content of leaves was found significantly higher in plants sprayed with EB5, EB9, and TC-9 as compared to leaves of control plants or plants sprayed with other endophytes. However, plants sprayed with TC-310 registered significantly higher total leaf chlorophyll content as compared to control and other treatments except EB9 and EB3 (Table 4).

## 4. Discussion

Bacterial blight, caused by *Xanthomonas axonopodis* pv. *punicae* is majorly controlled by chemical treatments, application of antibiotics such as streptocycline, or management practices such as stem solarization (Sharma et al., unpublished). However, due to excessive use of antibiotics, a resistant population of *Xap* has recently been reported [40]. Moreover, the Government of India has banned the production and usage of antibiotics; therefore, there is a pressing need for alternative, ecofriendly, and sustainable practices for blight management. Application of endophytes is one such approach which has the potential for biocontrol of pathogens [41].

Endophytes are microbes exhibiting a mutual relationship while residing inside the above- or below-ground host tissue. In the current study, a wide range of pomegranate host tissues: above-ground (leaf and stem) and below-ground (roots) parts of field-grown cultivars of pomegranate, as well as in vitro propagated explants (nodal segment and shoot tips), were explored for isolation of endophytes. Interestingly, tissues of the wildtype (*Nana and Daru*) yielded a lower population density of endophytes as compared to commercially cultivated varieties (Ganesh and Bhagawa). On the contrary, when some other wild and cultivated varieties of pomegranate were explored as a source of beneficial endophytes, the wild cultivar yielded more endophytes than two of the cultivated varieties [26]. However, the overall population density of the endophytes reported in that study was less than the density reported in the current study. This could be due to the differences in the regions from which the pomegranate varieties were obtained as the cultivars used in the previous study were grown in a colder state of India (Himachal Pradesh), while the varieties in the current study were from a hotter region (Maharashtra). Environmental factors such as host habitat or soil condition, genetic factors such as host genotype, and physiological factors such as age of the plant and tissue type have been reported to influence the density and diversity of endophyte recovered from plants [42,43,44]. Moreover, in this study, we isolated endophytes from field-grown and micro-propagated pomegranate explants of Bhagawa, a widely commercially cultivated variety. Endophytes, unlike artificial contaminants, can have a positive, neutral, or negative impact on the micro-propagation of the explant [45]. Despite their utility, not many reports are available on the isolation and utilization of endophytes from tissue-cultured plants [46], and none are available for pomegranate to the best of our knowledge. Thus, this study, for the first time, reports the presence and isolation of endophytes from micro-propagated pomegranate explants.

Furthermore, endophytes can have antagonistic effects on plant pathogens, and, to check this, the first step is to perform an in vitro evaluation against the pathogen. The literature is replete with examples where endophytes have been tested against pathogens in vitro. For example, endophytes isolated from pomegranate were tested against blight causing *Xap*, but the study was limited to an in vitro analysis only [26]. Similarly, several bacterial endophytes isolated from various plant sources were found to be effective in in vitro inhibition of bacterial leaf blight disease-causing *Xanthomonas oryzae* pv *. oryzae* in rice [47]. We screened bacterial endophytes isolated in the study against *Xap* on artificial media (NGA) and obtained a significantly high level of inhibition, with two isolates showing more than 50% inhibition of *Xap*. Furthermore, some of these effective endophytes also reduced the amount of fuscan produced by *Xap*. Fuscan is a pigment produced by some strains, and it has been suggested to be involved in pathogen aggressiveness [48]. The absence or reduced amount of fuscan produced by *Xap* under the influence of endophytes indicates the ability of endophytes to interfere with pathogen aggression.

To further confirm the antagonistic effect of endophytes on *Xap*, in planta assays were also carried out with the most effective isolates selected on the basis of results of the in vitro study. The results indicated that, when plants were primed by inoculating endophytes 8 days prior to challenge inoculation by the pathogen, the disease incidence and severity reduced significantly. Interestingly, our results also revealed that the isolate which showed the highest inhibition in vitro (TC-6) was not the best-performing isolate in planta. This indicates that the interaction between endophytes and pathogens may vary under in vitro and in vivo conditions; therefore, in planta antagonism evaluation studies must be carried out to validate the in vitro results.

While the inhibitory effect of endophytes on in vitro growth of *Xap* might be through the secretion of certain antagonistic compounds and secondary metabolites [49,50], the in planta inhibition of *Xap* by application of bacterial endophytes could be due to endophyte-mediated induced systemic resistance response (ISR). This priming of the host involves a higher induction of defense-related enzymes, namely *,* peroxidase, polyphenol oxidase, and phenylalanine ammonia lyase resulting in the higher accumulation of total phenols [24,49]. In the current study, we observed higher levels of phenolic compounds and leaf chlorophyll compound in endophyte-inoculated plants even after 90 days of inoculation, indicating a long-lasting effect of endophytes on the host.

These microbes play an important role in altering gene expression, metabolism, and physiological processes responsible for resistance reactions to both biotic and abiotic stresses in host plants [24]. There are also reports on the involvement of endophytes in inducing anatomical, physical, and biochemical changes in leaves of the host plants by altering cellulose content, leaf toughness, and lamina density. The significant impact of endophytes on the defense response of host plants against pathogens might be through early detection of pathogens by host plant cell surface receptor kinases, through cytoplasmic kinase-mediated intercellular responses, and by triggering of ethylene and jasmonic acid transduction pathways [24,50]. Zhao et al. [50] and Naveed et al. [51] reported the enhanced shoot and root growth, fresh and dry weight, and chlorophyll, phenol, protein, and cellulose contents of tissues, in endophytic microbe-inoculated plants. More detailed studies to unveil the exact mechanism via which endophytes antagonize *Xap* pathogen are required in future.

Application of endophytes as biocontrol agents is a promising approach toward sustainable management of diseases in economically important crops. Diverse microorganisms such as *Penicillium glabrum*, *Neofusicoccum parvum*, *Colletotrichum* spp., *Phomopsis* sp., *Nigrospora* sp., *Phyllosticta* sp. *Quambalaria cyanescens*, and *Bacillus subtilis* strain NS03 have been reported as promising endophytes in pomegranate [52,53,54,55,56,57]. Several other endophytic bacteria such as *Pantoea* sp., * Bacillus amyloliquefaciens* LE109, *B. subtilis* LE24, and *B. tequilensis* PO80 have been reported to suppress the *Xanthomonas* in rice and citrus plants [47,58]. In our study, endophytes belonging to the genus *Bacillus* were also recovered from field-grown and micro-propagated tissues. Moreover, these endophytes were found to successfully suppress the blight-causing pathogen in pomegranate. Similarly, *Bacillus subtilis* was also reported by many researchers as a potential and effective endophyte for ecofriendly plant disease management, which is in consonance with our findings [59]. Furthermore, it would be interesting to explore the effect of more than one efficient endophyte on blight incidence and severity in future. Therefore, the findings of the current study advocate for the prophylactic use of bacterial endophyte Bacillus subtilis along with other effective isolates for ecofriendly management of bacterial blight-causing pathogen *Xanthomonas axonopodis* pv. *punicae* in pomegranate.

## Figures and Tables

**Table 1:** Endogenous bacterial population size in various tissues of pomegranate.

Variety/Genotype	CFU Per Gram of Tissue			Number of Isolates
	Stem/Shoot Tip/Nodal Segments	Leaves	Roots	
Ganesh	8 × 10^7^ (EB1)	2.67 × 10^7^ (EB2)	7.33 × 10^7^ (EB3)	3
Bhagawa	2.16 × 10^7^ (EB9, EB11, TC 1, 2, 4, 6, 7, 9, 10, 130, 137, 310, A_2_B, B)	2.56 × 10^7^ (EB4, EB5, EB10)	3.6 × 10^7^ (EB6, EB7, EB8)	20
Nana	7 × 10^6^ (EB12)	-	-	1
Daru	5 × 10^6^ (EB14)	-	3.33 × 10^6^ (EB13)	2

**Table 2:** Effect of the bacterial endophytes on growth of *X. axonopodis* pv. *punicae* in dual culture and fuscan production by *X. axonopodis* pv. *punicae* (*Xap*).

Endophytes	Percent Growth Inhibition of *Xap* over Control *^,$^	Fuscan Production by *Xap*
EB1	31.58 ± 2.21 ^f,g,h,i #^	0
EB2	30.42 ± 0.42 ^h,i,j^	1
EB3	36.77 ± 2.02 ^e,f,g^	0
EB4	34.83 ± 1.82 ^f,g,h^	0
EB5	35.24 ± 1.42 ^f,g,h^	0
EB6	32.45 ± 1.42 ^f,g,h,i^	1
EB7	29.93 ± 1.92 ^h,i,j^	0
EB8	25.06 ± 0.48 ^j,k^	2
EB9	42.42 ± 1.48 ^d,e^	1
EB10	26.86 ± 1.62 ^i,j,k^	2
EB11	26.92 ± 0.90 ^i,j,k^	3
EB12	25.03 ± 0.97 ^j,k^	2
EB13	32.42 ± 1.89 ^f,g,h,i^	0
EB14	25.06 ± 0.48 ^j,k^	3
TC-1	15.65 ± 1.25 ^l^	3
TC-2	22.32 ± 2.28 ^k^	2
TC-4	51.12 ± 2.16 ^a,b^	1
TC-6	56.35 ± 2.66 ^a^	0
TC-7	37.91 ± 0.84 ^d,e,f^	2
TC-9	43.00 ± 0.32 ^c,d^	1
TC-10	29.53 ± 3.66 ^h,i,j^	2
TC-130	48.75 ± 2.20 ^b,c^	1
TC-137	48.61 ± 4.68 ^b,c^	0
TC-310	49.40 ± 4.14 ^b^	0
TC-A2B	49.84 ± 3.18 ^b^	0
TC-B	31.03 ± 1.95 ^g,h,i,j^	2
Control	0.00 ± 0.0 ^m^	3
CV (%)	10.78	--
SE (m)	2.12	--
SE (d)	2.99	--
Critical difference (*p* ≤ 0.05)	6.02	--

EB series of endophytes were isolated from different tissues of field-grown pomegranate plants, TC series of endophytes were isolated from explants of in vitro grown pomegranate plants. * The percentage inhibition data represent the mean of three replicates ± standard error. The values were arc-sin transformed. ^Letters^ Significance among treatment means once again checked and revised. ^#^ Values in the second column with the same letters are not significantly different. ^$^ The data reported here were recorded 8 days after inoculation.

**Table 3:** Identification of promising bacterial endophytes by 16S rDNA sequence comparison.

Isolate	Query Coverage (bp)	Identity (%)	Sequence Homology	Gene Bank Accession Number	NAIMCC * Accession Number
TC6	1426	100.00	*Bacillus subtilis*	KY575578	NAIMCC-B-03179
TC7	1414	99.08	*Burkholderia stabilis*	KY575579	--
TC130	1414	100.00	*Bacillus licheniformis*	KY575581	--
TC 137	--	--	*Bacillus subtilis*	OP999332	--
TC310	1424	99.51	*Bacillus tequilensis*	KY575582	NAIMCC-B-03180
EB4	1418	99.86	*Bacillus tequilensis*	KY575583	--
EB6	1427	99.09	*Lysinibacillus macroides*	KY575584	--
EB9	1420	99.93	*Bacillus subtilis*	KY575585	--

* National Agriculturally Important Microbial Culture Collection, Mau, India.

**Table 4:** Physiological and biochemical response of pomegranate plants upon endophytic bacterial colonization.

Treatment	Relative Leaf Water Content (%)	Total Phenol Content (mg Catechol Equivalent/100 g FW)	SPAD ^$^ Value	Total Leaf Chlorophyll (mg/g FW)
EB-3	85.87 ± 0.39	43.33 ± 1.76 ^b,c^	68.74 ± 2.37	2.23 ± 0.09 ^a,b^
EB-4	85.45 ± 0.89	44.00 ± 2.31 ^b,c^	66.31 ± 2.78	2.18 ± 0.07 ^b,c^
EB-5	84.48 ± 0.54	52.67 ± 8.51 ^a,b^	66.12 ± 2.90	2.11 ± 0.04 ^b,c^
EB-9	85.88 ± 0.64	55.33 ± 5.81 ^a,b^	68.24 ± 3.70	2.45 ± 0.13 ^a^
TC-6	86.09 ± 1.44	45.33 ± 3.33 ^b,c^	60.64 ± 1.55	2.10 ± 0.07 ^b,c^
TC-9	83.99 ± 1.71	61.33 ± 2.67 ^a^	63.93 ± 2.45	2.18 ± 0.03 ^b,c^
TC-310	86.09 ± 1.44	35.33 ± 2.40 ^c^	66.13 ± 1.52	2.28 ± 0.10 ^a,b^
Control	80.66 ± 1.68	36.00 ± 2.31 ^c^	57.36 ± 1.49	1.96 ± 0.08 ^c^
CV (%)	2.45	15.74	6.58	6.39
SE (m)	1.20	4.24	2.46	0.08
SE (d)	1.68	6.00	3.48	0.11
Critical difference (CD) at *p* ≤ 0.05	NS	12.83	NS	0.24

Data represent the mean of three replicates ± standard error of the treatment mean; NS, nonsignificant. Values in the same column with the same letters are not significantly different. ^$^ Soil Plant Analysis Development.

## Data Availability

The data that support the findings of this study are openly available from GenBank NCBI at https://www.ncbi.nlm.nih.gov/ (accessed on 28 September 2022), reference numbers KY575578, KY575579, KY575581, KY575582, KY575583, KY575584, and KY575585.

## Supplementary Materials

The following supporting information can be downloaded at https://www.mdpi.com/article/10.3390/microorganisms11010005/s1: Figure S1. Confirmation of the identity of the pathogen isolated from artificially inoculated plants as Xap based on the presence of the 491 bp amplicon obtained using gyrB-specific primers for PCR amplification.
