# Supplementary material for: In Vitro and In Planta Antagonistic Effect of Endophytic Bacteria on Blight Causing Xanthomonas axonopodis pv. punicae: A Destructive Pathogen of Pomegranate
Source: Microorganisms. 2022 Dec 20;11(1):5. doi: 10.3390/microorganisms11010005 (PMC9860609; doi:10.3390/microorganisms11010005)
Supplement: Supplementary file 1 [file microorganisms-11-00005-s001.zip › microorganisms-1985193-supplementary.pdf]

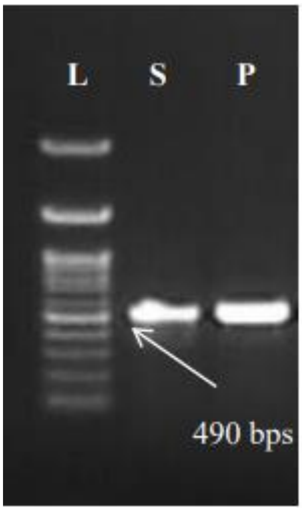

Supplementary Figure S1. PCR amplification of *Xap* DNA from pure culture and symptomatic leaf tissue using *gyrB* specific primers (S: DNA from symptomatic leaf tissues, P: DNA from pure culture of *Xap*)
